# Effects of gamification on the engagement and translation proficiency of the Chinese EFL learners: a case of Quizizz

**DOI:** 10.3389/fpsyg.2026.1813663

**Published:** 2026-04-13

**Authors:** Juan Zhang

**Affiliations:** School of Foreign Languages, Guangdong University of Technology, Guangzhou, China

**Keywords:** classroom interaction, engagement, gamification, gamified language learning and teaching, Quizizz, translation education, translation proficiency

## Abstract

Gamified learning and teaching have gained their grounds in education across various levels and disciplinary fields. Research has demonstrated its effectiveness in enhancing learning outcomes and facilitating education. However, in the field of language learning, particularly translation education and training, gamification has not been used as widely as in other disciplinary areas. The present study addresses this issue and experiments by using one of the popular gamification systems named Quizizz in the translation classroom to improve both the engagement and outcome of students' translation proficiency. A quasi-experiment method was used and the effectiveness of using Quizizz was examined by comparing the engagement and achievement in the translation classroom between an experiment group using Quizizz, and a control group receiving traditional, non-gamified instruction. The results of the analysis showed a positive influence of using Quizizz in improving the translation proficiency of the learners, whereas no significant difference was found in terms of engagement between the Quizizz group and the traditional non-gamified group. In light of the results, implications were discussed regarding the empirical directions and pedagogical practice.

## 1 Introduction

Gamification has attracted increasing attention in the field of education, with its applications expanding across various educational context in recent years. It is the integration of game elements into pedagogical settings, such as the features, mechanics, aesthetics, and design of games to engage learners and promote learning (Alsawaier, 2018; Kapp, 2012; Almeida and Simóes, 2019). Compared with non-gamified course design, learners taught under the gamified teaching mode could be more motivated inside and their inherent interest could be aroused, leading to greater engagement. Due to a range of unique game elements and mechanics integrated into learning design, such as a reward system, storyline, competition, interaction, timely feedback, etc., learners of almost all educational levels could feel motivated and empowered, ultimately enhancing the actual learning outcome (Kiron and Vassileva, 2018; Manzano-León et al., 2021).

The effectiveness of gamified learning has been shown by studies that involve students learning different subject areas at various educational levels. The benefits of gamification in education include greater learner involvement in projects, skill development and application in real contexts (Almeida and Simóes, 2019). Seixas et al. (2016) found the effectiveness of gamification in promoting engagement of elementary school students in Brazil, mainly featured in the use of a reward system. The meta-analysis conducted by Huang et al. (2020) also confirmed such a positive influence of gamified learning on the learning outcomes across various subject areas and educational stages, with a small to medium effect size.

The trend of using gamification also emerges in the context of language learning. However, its application in language classrooms remains relatively limited as a new field of research (Dehghanzadeh et al., 2019). The validity and effectiveness of gamified language learning require substantial support from empirical studies. Solid research efforts are needed to examine the impact of gamification on the acquisition of various language skills and to evaluate the achievement of language learning (Rueckert et al., 2020). Its application in the domain of translation education or translation learning is even scarcer. Therefore, this study aims to investigate the utility of Quizizz, as one of the gamification applications, in the instruction of translation skills of EFL learners in China.

## 2 Related works

### 2.1 Gamifying the language classroom

Among the studies examining the use of gamification in language learning and teaching, most of them have shown its positive affordances in language education. Such a learning mode has been supported by studies to have its beneficial effects on psychological and affective states, as well as cognitive performances. It was also found to promote motivation, willingness to communicate, and hence language learning outcomes (see the review by Hung et al., 2018). A gamified EFL (English as a Foreign Language) class was found to be more conducive for the brain to process and retain information, leading to greater learner autonomy (i.e., a greater sense of agency) than traditional education mode (Rueckert et al., 2020).

With a focus on examining the effect of a game-based mode on the affective, cognitive, and motivational performances of language learners, Hwang et al. (2017) found that the game-based approach used in the listening classroom could improve their listening achievement and motivation, but did not decrease anxiety level. Nevertheless, this learning approach particularly benefited the students with higher levels of anxiety in learning and gaming behaviors, as well as their listening achievement.

Slightly different from part of the findings of Hwang et al. (2017), the quasi-experimental study by Hung (2018) found that gamifying the flipped classroom was beneficial for EFL university students due to its effects in reducing learner anxiety and enhancing their motivation to speak in class. The researcher noted that measuring learning outcomes, for example, through achievement tests, was needed to provide a more complete understanding of the effects of gamifying the flipped classroom.

Gamified language learning is also perceived and evaluated positively by learners and teachers in language learning. In the study by Zou (2020), which examined the perceptions of learners and teachers toward a flipped gamified language learning program, both learners and teachers perceived such a mode as positive and evaluated it as increasing the motivation and engagement, developing learning skills and confidence, and improving learning outcomes. The mode used in the study was the gamified flipped language classroom. It could be possible that the perceived effects resulted from a combination of the two factors: gamification and flipping.

As shown above, gamified learning has been applied in the language classroom and its benefits in promoting language skills, ranging from listening, speaking, pronunciation, writing, grammar and vocabulary learning, have been examined. However, compared with vocabulary learning as one of the language competence components, the studies focused on other skill areas are still less common (see the review by Hung et al., 2018). Furthermore, inconsistent findings as to the actual effects of gamification on improving learning outcomes warrant further extensive studies. For example, in a longitudinal study conducted over 16 weeks, Hanus and Fox (2015) found that students learning in the gamification mode turned out to be less motivated, less satisfied and less empowered than students learning in the non-gamification mode. Similarly, Rachels and Rockinson-Szapkiw (2017) found no significant difference in Spanish learning achievement and self-efficacy between the gamification group and the traditional learning group. Another issue is that most of the studies on gamification in language learning and teaching have not considered the engagement of language learners. Additionally, within the scope of knowledge of the author, translation skills have not yet been examined in the context of gamified learning.

### 2.2 Engagement in language learning

Engagement indicates learners' invested efforts in learning, which is often related with better academic achievement and persistence in learning. According to researchers (e.g., Hiver et al., 2021; Oga-Baldwin, 2019), engagement is featured in the notion of action, including both the quantity and quality of active involvement and commitment to learning. Another key feature is its dependence on context, such as the specific cultural and environmental contexts (Fredricks et al., 2004). Engagement is also object-specific, that is, it is closely related with a certain topic, person, situation, or an activity and task. Researchers generally conceptualize engagement as a multi-faceted construct (Aubrey et al., 2020; Hiver et al., 2021), consisted of behavioral, cognitive, emotional, and social aspects. Behavioral engagement refers to the extent to which learners actively participate in and invest efforts in learning activities. Cognitive engagement involves the aspect of learners' mental efforts in learning. Emotional engagement refers to learners' affective reactions when they are engaged in their learning activities or tasks, and positive affective reactions are regarded as ideal emotional engagement. Social engagement refers to the active interactions of learners with others.

Related studies of engagement in language learning setting mostly centered around underlying conditions or factors that makes it happen or increase, or the opposite direction that makes disengagement appear or decrease. As aligned with most of the findings and views from general education and other disciplinary subjects, it was generally found that engagement was usually elicited by classroom-related contexts and learner's traits. Features and conditions external to language learners have been examined extensively, including teacher support and practices (Hoi, 2022; Hu and Wang, 2023; Sadoughi and Hejazi, 2021; Zhou et al., 2023a); classroom or group climate (Aubrey, 2022; Derakhshan et al., 2024; Wang et al., 2025; Yang and Liang, 2025), authentic tasks (Benito Cox and Montgomery, 2019), familiar and relatively easier task topics (Aubrey, 2022), factors internal to language learners were also documented to influence engagement, usually functioning as mediating factors, including language proficiency (DeWaelsche, 2015; Huang et al., 2017), motivations (Aubrey et al., 2020; Zhou et al., 2023b), expectancy and task value belief (Hoi, 2022), self-efficacy, mastery and performance goals (Liu et al., 2022), anxiety (Wang et al., 2023), psychological needs satisfaction (Zhou et al., 2023a), grit (Sun et al., 2024), task orientation (Zhong and Zhan, 2024). Engagement is negatively impacted by boredom (Derakhshan et al., 2024), anxiety (Li et al., 2024) and extrinsic motives (Oga-Baldwin and Nakata, 2017). It was also found to have reciprocal effects with factors such as task value beliefs (Vo et al., 2025), language learning enjoyment (Guo, 2021), perceptions of teacher motivational practice (Guo et al., 2023).

Engagement has also been studied by examining it in different classroom settings, with technology-enhanced teaching as one of the typical cases, including language classroom with Web 2.0 applications (Liu et al., 2016), mobile application (Cho and Castaneda, 2019), flipped classroom (Amiryousefi, 2019), immersive virtual reality environment (Lee et al., 2024), AI-empowered classroom environment (Xu and Li, 2024), AI-driven chatbots (Wang and Xue, 2024), and ChatGPT for text revision (Koltovskaia et al., 2024), etc. Eltahir et al. (2021) examined Arabic language learners' engagement in their grammar course which used Kahoot! as an online formative assessment tool. The study revealed more gains of using the application in promoting learners' motivation and mastery of grammar knowledge, compared with the traditional teaching method. However, the impact on their engagement was not clearly shown due to lack of comparison with a control group. Cho and Castaneda (2019) also examined engagement in L2 grammar classroom with the design of game-like activities and found this class feature facilitated motivational and affective engagement. Although these two studies focused on using gamification or game-like activities in grammar learning of language, the impact on engagement was not well evidenced due to either a lack of comparison with a control group or lack of measurement in cognitive and social engagement. As suggested by Yu et al. (2022), serious games or gamified learning should be developed and applied in language classroom to promote learners' engagement. However, empirical research focused on investigating the effects of gamification application in language classroom is noticeably lacking.

### 2.3 Research on translation training and translation competence

Translation is one of the essential skills for language learners of higher-proficiency levels. In post-secondary educational contexts, translation courses are usually given as an introductory course, with limited instructor-learner interactions, potentially reducing learner motivation and engagement (Parvaresh et al., 2018). Based on their investigation of the resistance and lack of motivation among translation students, the researchers further argued for the use of technology to facilitate translation education for better acceptance and learning outcomes.

Despite the widespread use of educational technologies to facilitate the learning and teaching practice in other disciplines, particularly in contrast to the extensive technological applications in its counterpart area, that is, language learning or acquisition, technology-based translation education has not been in common practice. Given the increasing importance of technology in academic fields, its applications and potential affordances in facilitating translation education should be considered (Jiménez-Crespo, 2017). However, research examining the use of gamification systems such as Quizizz in translation training remains scarce. Integrating gamified learning into translation instruction may increase classroom interaction, motivation, and learning outcomes, given its effectiveness that has been shown by studies done in other disciplinary areas of learning.

Translation training research has recently focused on the translation process, translation product assessment, and translator factors related to translation competence or performance (Latif, 2020). As for the teaching practice of translation training, although studies have been conducted on the topics such as feedback provided in translation class (Washbourne, 2014; Alfayyadh, 2016), both the scope and depth of research need to be further expanded and strengthened. Motivational variables of translation students such as students' engagement in the translation training or classroom, remain under-investigated among related studies.

As one important aspect in translation training, translation competence has been widely studied, particularly as to its components and effective measurements of translation competence. Different models have been developed by researchers (for example, Neubert and Gregory, 1992; Orozco and Hurtado Albir, 2002; PACTE, 2003; Pym, 2003). Among these different propositions, several similar components, such as bilingual communicative competence, instrumental competence, a translator's psycho-physical disposition, are identified as the essential elements of translation competence. In order to fully develop these skills, interactive and dynamic learning contexts are needed, particularly through means of technological utilization such as gamification systems. Merely using the transmissionist teaching approach in teaching translation skills cannot achieve this end.

Another issue with translation training studies is that they should examine different instructional techniques with the use of more rigorous designs (Latif, 2020). It was contended that translation training effectiveness should be assessed in terms of translation competences, given that a large number of translation training studies generally assessed trainees' perceptions.

According to the existing literature, translation quality can be assessed by several methods, including intuitive assessment, error analysis, corpus-based evaluation, rubric scoring, mixed-methods scoring, item-based assessment, and comparative judgement (Han, 2020), with their respective shortcomings and strengths in measuring the translation quality. Rubric scoring is a relatively more reliable and practical method among those methods with high criterion-related validity (Waddington, 2001). In our study, a rubric scale is adopted to assess the students' performance in the two rounds of translation tests as the objective measurement of their translation proficiency.

### 2.4 Quizizz app as an educational gamification tool

**Figure 1:**
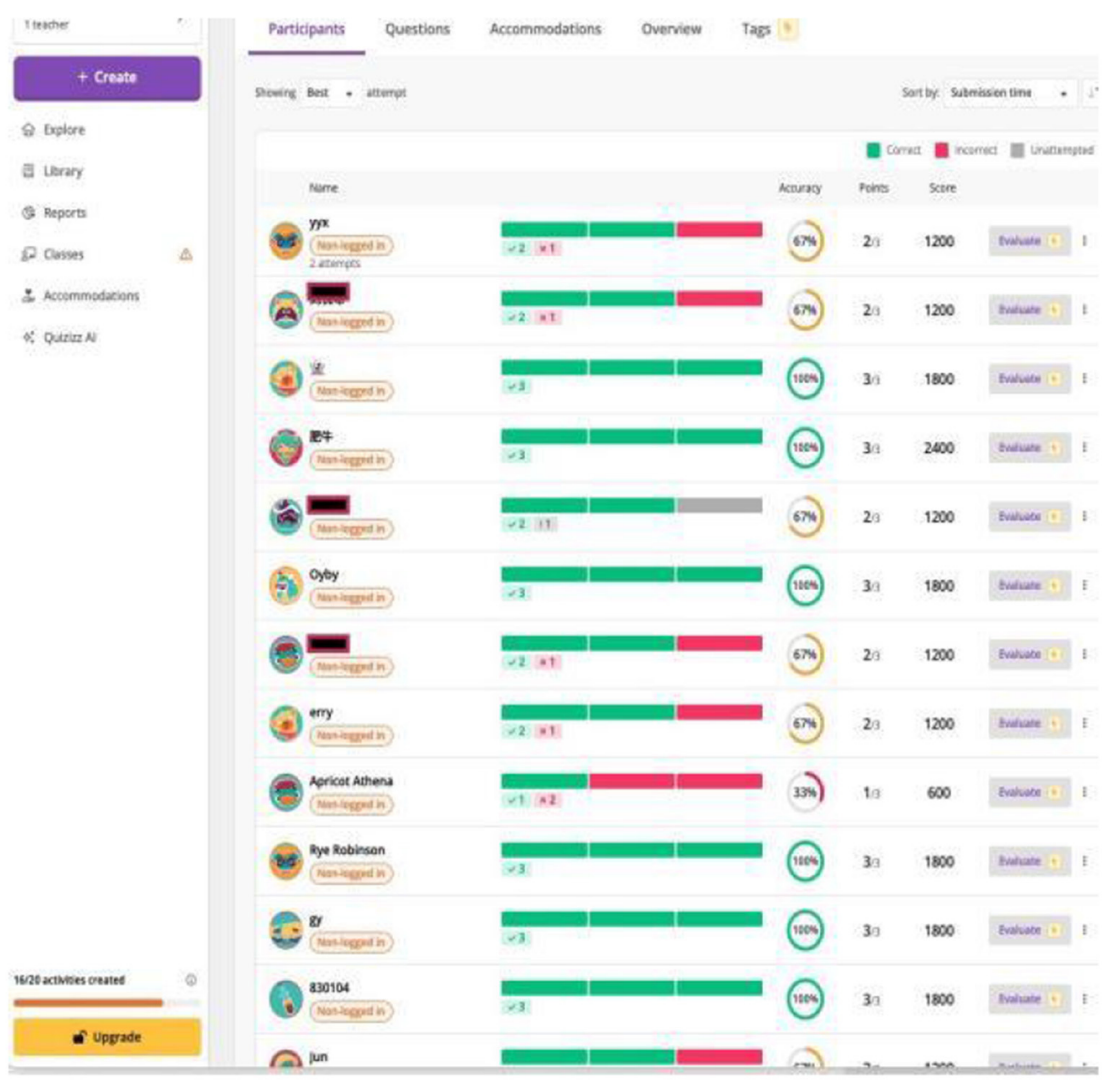
Screen capture of activities in Quizizz.

Quizizz is a web-based online gamified educational application. With Quizizz, small quizzes and questions can be created by instructors and in-class discussions can also be initiated for the students to express their opinions on learning-related questions. Quizizz has gamification elements such as leader boards, time limits, points, and social relatedness, all of which enhance learners' participation and sense of empowerment in completing learning tasks (Yunus and Hua, 2021). Yunus and Hua (2021) also confirmed its positive effect on enhancing the learning of the irregular English verbs among the Malaysian primary school learners. Figure 1 shows the example of task completion results on Quizizz which was used for the purpose of completing tasks in class.

Many studies using Quizizz as an intervention in the language classroom examined the perceptions and acceptance of its use among learners and teachers. Halim et al. (2020) examined the use of two online quiz games, Quizizz and Kahoot!, in primary school ESL lessons and found positive perceptions and acceptance among learners. The review by Lim and Yunus (2021) also showed that teachers had positive attitudes toward integrating Quizizz in ESL classroom and perceived it as both effective and feasible in promoting learning and motivation. However, most related studies regarding the use of Quizizz in language classrooms have focused on perceptions and attitudes rather than its actual benefits and impact on language competence development.

Although the use of gamification tools has been shown by some studies to contribute to better learning achievement in several skill areas, such as vocabulary acquisition (Yu, 2014), communicative competence (Agbatogun, 2014), and writing proficiency (Yu and Wang, 2016), most of these studies used Clicker which is similar to Quizizz as one of the gamification applications. Studies reporting impacts of utilizing Quizizz in language learning have not been extensively carried out, particularly for its application in promoting translation proficiency of language learners.

Given the limited scope of research focused on motivational variables in translation training, and the limited application of technology in translation education, this study aims to investigate the effects of using Quizizz on translation proficiency and engagement among EFL learners in the translation classroom. To be specific, our study seeks to answer the following research questions:

1. What is the effect of using Quizizz on improving translation proficiency among Chinese EFL learners?
2. What is the effect of using Quizizz on improving engagement in translation class among Chinese EFL learners?

## 3 Methods

### 3.1 Participants

A total number of 117 second-year undergraduate students from a Chinese university were recruited for the study. They all majored in Business English and took the compulsory course of Translation between English and Chinese during their third semester at the university. The sample consisted of 29% male students and 71% female students, with an average age of 19 and an average of 11 years of learning English. Ethical approval was obtained from the Ethics Review Committee of the university where the study was conducted. All participants signed the written informed consent before the study started and they were told that they would be able to withdraw from the study at any time.

All participants had similar formal English language learning experience, as English was a mandatory subject since primary school and also one of the required subjects in their university entrance examination. Moreover, they had similar English proficiency level because they had to meet the baseline English proficiency score before they were admitted into the university. They took part in the study on a voluntary basis.

### 3.2 Design of the study

A pretest-posttest quasi-experimental design was used in the study. The participants were assigned to either an experimental (*n* = 53) or a control group (*n* = 52) based on their existing class allocations. The two intact classes had similar academic backgrounds and they have not attended any translation expertise training or other translation courses before the study. However, the translation pretest mean scores differed between the two classes (Control: M = 146.10; Quizizz: M = 156.58), as indicated by the results of the translation pretest (see Table 2). The two groups were taught by the same instructor, who had a language teaching experience of 12 years. As random assignment was not feasible in this study due to academic administration and course arrangement, potential selection bias may not be completely ruled out. In the data analysis, ANCOVA was therefore employed to statistically control for baseline differences.

At the outset of the course, the participants were asked to complete the engagement questionnaire. In the first session of their course, the translation pre-test was administered. For the experimental class, the participants received a brief training on how to register and use Quizizz in class. From the second session on, the intervention started by using the Quizizz in the routine course of the experimental group (See Table 1 for the intervention details). All the teaching procedures were identical for the experimental and control groups except that PowerPoint slides, instead of Quizizz, was used in the teaching of the control group. Quizizz was used exclusively during in-class sessions and not used as homework assignment outside class. The same task items were used in both groups to ensure content equivalence. The only difference was the mode of response, that is, gamified vs. verbal discussion. At the last session of the course, the participants took the translation posttest and completed the second round of engagement questionnaire before they exited the study.

**Table 1:** Intervention operation details.

Component	Description
Duration	12 sessions (Week 2–13)
Items per session	5–8 multiple-choice questions
Time limit for each session	15–30 min
Gamification features	Leaderboard, points, instant feedback
Power-ups	Disabled
Rewards	Disabled
Outside-class use	No
Total exposure	~60–80 items

In order to ensure the validity and reliability of the study, several measures have been taken. First, the interval between the pretest and the posttest of the engagement scale was controlled at an interval of 14 weeks, so as to minimize the memory effects. Second, the engagement questionnaire and translation tests were adapted from validated instruments with high reliability and validity as reported by previous studies. They were also reviewed by two experts respectively in motivation and translation studies. Furthermore, the reliability of the measurement instrument and test scores was calculated and reported in the following section.

### 3.3 Measures and translation tests

A pretest of translation proficiency was given to all the participants at the outset of the study. A paralleled translation post-test was given upon completion of the study at the end of the semester. In both tests, the participants were asked to translate an English passage into Chinese, with a length of 143 words for the pre-test and 167 words for the post-test, and a Chinese passage into English, with a length of 272 words for the pre-test and 266 words for the post-test (see **Appendix I** for the two tests).

The translation tasks in the two tests were adapted from the translating practice part of Level 3, China Accreditation Test for Translators and Interpreters (CATTI), which is a nationally recognized translation qualification test. The Level 3 test evaluates intermediate level of translation competence. Competencies required in Level-3 test include the testee's capability of expressing the original content with the correct use of grammar and rendering the translated texts in a meaningful and contextually appropriate way. The test aligns with the international translation qualification tests such as NATTI and it aims at evaluating the translators' competence required for professional competences who intend to work in the fields of international business, inter-cultural exchanges and communications. In order to ensure parallelism of the two tests, the lexical density, syntactic complexity, and genre features of the pre- and post-tests were evaluated by two instructors of translation course who had more than 10 years of experience in translation teaching. Both pre-test and post-test used in our study lasted for 90 min for the participants to finish their translation in the lecture time.

The scoring rubrics developed by Khanmohammad and Osanloo (2009) was used in scoring the translation tasks in the two tests due to its relative objectivity and detailed scales of assessment criteria (see **Appendix III**). Each test included two tasks: English to Chinese and Chinese to English, with each task equally weighted as 100 points, making a total possible score of 200. In the data analysis, the aggregated scores of the two tasks were analyzed as the total score. Two raters who had expertise in English teaching and translation studies independently scored the tasks. The raters were blind to both group assignment and test time. The inter-rater consistency, calculated by the Intraclass Correlation Coefficient (ICC, two-way random, absolute agreement) was 0.825 for the pretest and 0.856 for the posttest, indicating good consistency between the two raters. When there was a discrepancy in scores, the two raters discussed until they agreed on the final score. The translation proficiency test demonstrated a high level of reliability, with Cronbach's Alpha values of 0.906 and 0.868 respectively for the pretest score and the post-test score.

In order to measure the participants' engagement in the translation class, a questionnaire adapted from Elmaadaway (2018) was used (see **Appendix IV** for the details of the questionnaire). Engagement was defined as the passion and emotional involvement in participating and completing activities in learning (Skinner and Belmont, 1993). However, there has been various views among researchers as to the definition and dimensions of the engagement construct. As is widely recognized in related studies, our study adopted the dimensions put forward by Fredricks et al. (2004), which posits that engagement includes three different dimensions in literature: behavioral engagement (active participation in learning activities and positive attitudes toward learning), emotional engagement (affective and emotional reactions while participating in learning activities and tasks, such as interest, happiness, wellbeing, disgust, anxiety and frustration) and cognitive engagement (mental efforts of learners in understanding the learning content and reaching the highest levels of comprehension). The questionnaire by Elmaadaway (2018) covers the items of all the above dimensions and therefore, it was chosen as the measurement of engagement in this study.

The questionnaire consisted of 22 items, covering three dimensions: behavioral engagement (8 items), cognitive engagement (7 items), and emotional engagement (7 items). Responses were rated on a 5-point Likert scale ranging from 1 = “strongly disagree” to 5 = “strongly agree” (see **Appendix IV** for the questionnaire). The questionnaire was reported to have high validity and reliability by existing studies. In this study, the Cronbach's α values of the pre- and post-intervention measurement were 0.938 and 0.962, respectively, indicating its appropriateness for assessing engagement in translation learning.

### 3.4 Instructional design

The translation course lasted for 14 weeks, with one lecture session each week for 90 min. The course aimed at developing fundamental translation skills between English and Chinese. By the end of the course, students were expected to achieve translation competence in terms of accuracy, fluency and authenticity. Framed within the translation competence (TC) model developed by Hurtado Albir (2015), this course focused on developing the following three translation sub-competences of the model: methodological and strategic competences, contrastive competences, and translation problem-solving competences. All the three components involve both declarative and procedural knowledge, but more leaning toward the acquisition of procedural knowledge. As one of the essential pre-conditions to achieve the above purposes of our translation course, learning task design was given full consideration in planning and implementing the course. In each course session, the teaching focused on one topic or specific aspect of the three essential sub-competences (see **Appendix II** for the instruction schedule). In all, nine topics were covered as the teaching plan of the course.

The typical teaching steps include two parts: part One is the instructor's presentation of the targeted topics and skills, followed by the tasks for the students to answer questions raised by the instructor related to the topic or skill presented; Part Two is the translation workshop activities in which students did translation practices and discussed the strengths and weaknesses of their translation as well as challenges they had in their translation. The instructor gave feedback, comments, and suggestions in this step.

The intervention of using Quizizz was conducted in the first part of each teaching session, that is, when the instructor proposed questions related to the topic covered in that session. This step worked as the connecting stage between the instructor's presentation on the topic in each session and learners' practice of applying what they learned into their translation. On the one hand, choice-making tasks were easier than translation tasks for the students to complete so that this step could make students better geared toward the next step of translating practice. On the other hand, the students could be provided with appropriate scaffolding on both their awareness and skills in using what they learned into their translating practice. In order to effectively acquire and reinforce relevant skills and knowledge, Quizizz was used due to its effects as shown in previous research focused in other domains or skill areas.

**Figure 2:**
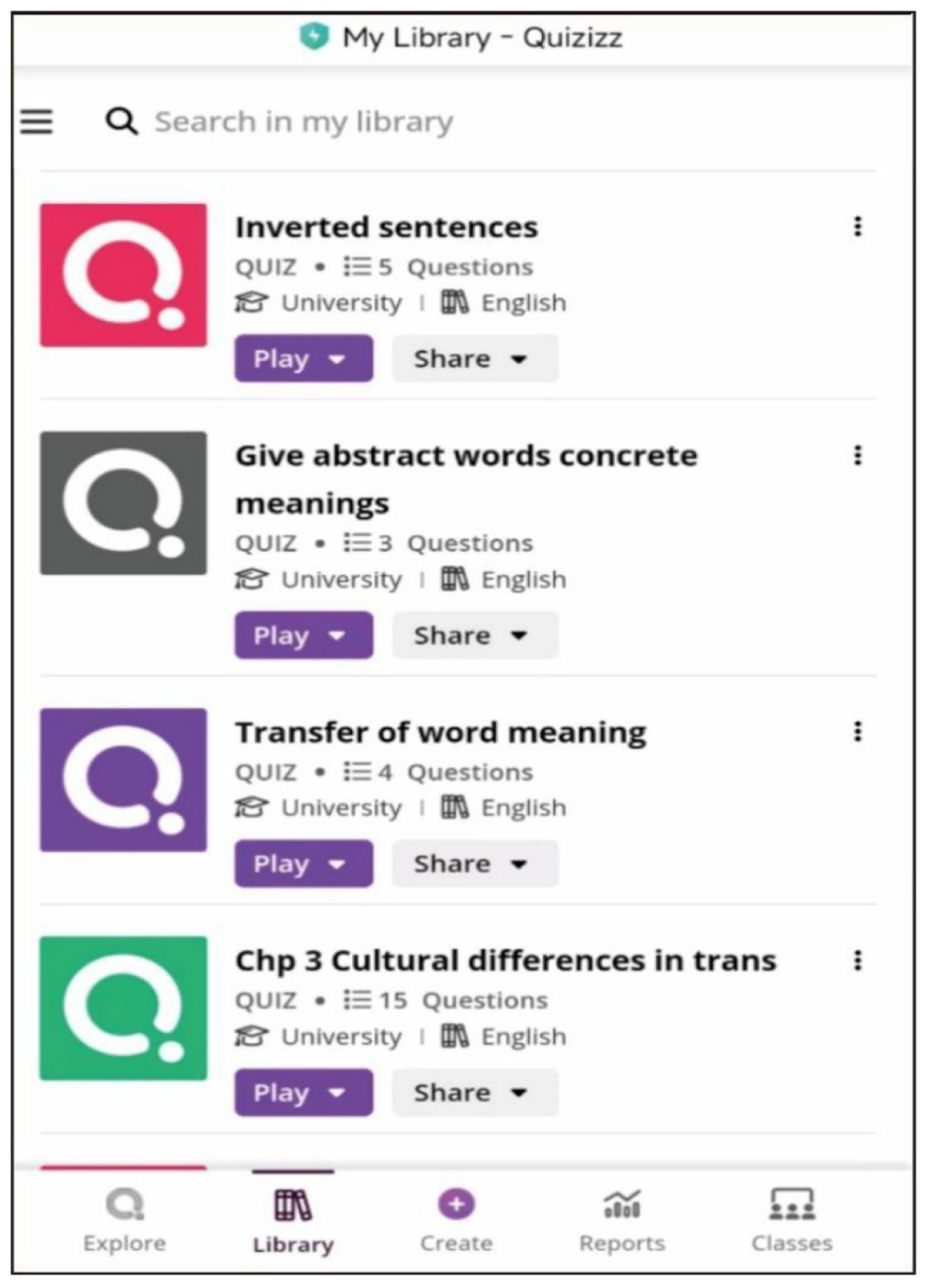
Screen capture of learning tasks in Quizizz.

The students in the experimental class were asked to give their answers by using Quizizz and then discuss their answers in class to check out their understanding and learning of the presented part. Figure 2 shows an example of learning tasks imported into Quizizz platform which was used in the experimental class. In the course session on the inversion, as one of the frequently-used translation strategies and techniques between the English and Chinese, the participants in the experimental class were asked to complete five multiple-choice questions on Quizizz app by using their mobile phones. Each of the questions include a sentence to be translated into the target language, followed by two or more possible choices for the students to select from as the optimal translation. The choice items were designed to ensure that the students had a good understanding of the strategies and techniques they had just learned so that they were able to transfer their understanding into appropriate use in producing translation in good quality. They were reminded by the instructor that when making their choices between the two translations, they needed to use what they learned from the instructor's presentation and explanation in the previous learning step of the lesson. After they finished the tasks, the instructor had discussions with the whole class on the answers of all the choice-making tasks. The students were encouraged by the instructor to provide the reasons for making their choices and ask any question they had about the tasks. As the last step, the instructor made a summary by revisiting how the strategies and techniques could be used in an appropriate way to produce translations in better quality.

For the control group, all the teaching and learning procedures were the same with the experimental class, with the only difference being that PowerPoint slides were used to show the same questions and the students answered the questions in verbal discussions only.

### 3.5 Data analysis

After excluding dropouts and incomplete responses, data from 105 participants were retained and analyzed using SPSS Version 25. The Kolmogorov-Smirnov tests showed that all variables were normally distributed, with *p* values for translation pretest, translation posttest, engagement pretest, and engagement posttest at 0.086, 0.200, 0.161, and 0.081, respectively (all > 0.05). Assumptions of linearity and homogeneity of regression slopes were also tested and met prior to conducting further analysis. Levene statistics of the pretest and posttest scores of both the engagement questionnaire and the translation competence did not reach the significant levels and the interaction effects between the pre-test and post-test engagement scores and translation competence scores did not reach the significant levels, either.

To examine the effects of using Quizizz in translation class on the engagement and translation proficiency, two one-way factorial analyses of covariates (ANCOVAs) were conducted. In the first analysis, group (experimental vs. control) was the independent variable, the translation posttest score was the dependent variable, and the translation pretest score served as the covariate. In the second analysis, the same procedure was followed, with engagement posttest scores as the dependent variable and engagement pretest scores as the covariate. These analyses were designed to control for baseline differences and isolate the effect of the intervention.

## 4 Results

### 4.1 Effects of Quizizz on translation proficiency

According to the descriptive statistics, the means and standard deviations of the translation post-test scores for the experimental group which used Quizizz were 169.55 and 6.10, respectively. The control group, which did not use Quizizz, scored 150.94 and 7.33, respectively. As shown in Table 2, the adjusted mean for the experimental group (168.67) was also higher than that of the control group (151.84).

**Table 2:** Descriptive statistics of translation pre- and post-test scores.

Group	Pre-test			Post-test (unadjusted)		Post-test (adjusted)	
	*N*	Mean	SD	Mean	SD	Mean	SE
Control	52	146.10	14.257	150.94	7.33	151.84	0.93
Quizizz	53	156.58	10.642	169.55	6.10	168.67	0.92

Covariates appearing in the model are evaluated at the following values: pre-test score = 151.3905.

The ANCOVA results for translation proficiency test scores are shown in Table 3. There was a significant difference in the translation post-test scores between the two groups after controlling for the impact of the pre-test scores with *F* = 153.152, *p* < 0.001), partial η^2^ = 0.600, 95% CI [0.48, 0.68]. This indicates that the experimental group, with the use of Quizizz in the translation classroom, had better achievement in translation learning than the control group which followed the traditional teaching method without using Quizizz.

**Table 3:** Results of ANCOVA of translation post-test scores.

Source	SS	*df*	MS	*F*	Sig.	η2
Pretest (covariance)	463.650	1	463.650	11.243^**^	0.001	0.099
Group	6,315.730	1	6,315.730	153.152^***^	0.000	0.600
Error	4,206.309	102	41.238			

^**^p < 0.01.

^***^p < 0.001.

R Squared = 0.694 (Adjusted R Squared = 0.688).

### 4.2 Engagement of the participants

According to the descriptive statistics provided in Table 4, the means of the pre-survey score of the Quizizz group (79.415) were slightly lower than that of the control group (79.846) before the intervention. However, after the intervention, the adjusted means of the post-survey score of the Quizizz group (82.191) were higher than that of the control group (81.383). This suggest that participants of both groups had their engagement levels improved at the end of their course. It is noteworthy that the Quizizz group, which initially had a lower engagement level than the control group, turned out to have higher engagement levels after the intervention.

**Table 4:** Descriptive statistics of engagement pre- and post- survey scores.

Group	Pre-survey			Post-survey (unadjusted)		Post-survey (adjusted)	
	*N*	Mean	SD	Mean	SD	Mean	SE
Control	52	79.846	8.445	81.539	11.067	81.383	1.129
Quizizz	53	79.415	8.906	82.038	9.296	82.191	1.118

Covariates appearing in the model are evaluated at the following values: Pre-survey = 79.6286.

However, further analysis revealed that the difference in post-survey engagement scores between the two groups did not reach the significant level, with *F* = 0.259, *p* > 0.05, partial η^2^ = 0.003, 95% CI [0, 0.06], as shown in Table 5.

**Table 5:** Results of ANCOVA of pre- and post-survey scores.

Source	SS	*df*	MS	*F*	Sig.	η^2^
Pre-survey score (covariance)	3,983.555	1	3,983.555	60.131	0.000	0.371
Group	17.129	1	17.129	0.259	0.612	0.003
Error	6,757.292	102	66.248			

R Squared = 0.371 (Adjusted R Squared = 0.359).

To further investigate changes in engagement levels, paired sample *t*-tests were conducted for both the experimental and control groups. As presented in Table 6, the engagement level of participants in the control group did not significantly change (*p* = 0.145 > 0.05). However, the engagement level of participants in the experimental group significantly increased after the intervention (*p* = 0.032 < 0.05), though the effect size was small (Cohen's *d* = 0.302). Although a small within-group difference was observed in the Quizizz group, the absence of a significant between-group difference suggests that no clear treatment effect on engagement can be established.

**Table 6:** Paired-sample *t*-test for engagement.

Group	Engagement	Mean	*N*	SD	*t*	Sig	*d*
Control	Pre-survey	79.846	52	8.445	−1.482	0.145	0.205
	Post-survey	81.539		11.068			
Quizizz	Pre-survey	79.415	53	8.906	−2.198	0.032	0.302
	Post-survey	82.038		9.296			

## 5 Discussions

Previous studies on the effectiveness of gamification have indicated that gamification applications could be facilitative in improving the outcome and performance of learners in a variety of subject areas. However, Quizizz, as an educational application, has not been extensively studied in L2 or foreign language classrooms, particularly in translation training. The present study aims to examine the effects of Quizizz on the translation proficiency and engagement of EFL learners in their translation class. In this study, the quasi-experimental method was used in which one group of participants learned translation skills with the use of Quizizz in their class time, while the other group of participants received instruction without the use of Quizizz. The following section is to elaborate on the discussions and implications based on the results shown in the previous section in response to the research questions proposed.

### 5.1 Comparison of learning gains in translation proficiency between the two groups

With respect to research question 1, in terms of the translation proficiency, students in the experimental group which used Quizizz in class significantly outperformed those in the control group which used conventional multi-media PowerPoint slides. This indicates that the use of Quizizz in the translation teaching classroom was effective in improving the students' translation proficiency. These findings align with research on gamification used in language classrooms, which suggest that gamifying language learning could effectively promote learning outcomes, and is, in general, positively perceived by learners and teachers to play important roles in leading to better learning outcomes (Halim et al., 2020; Lim and Yunus, 2021; Sénécal et al., 2022; Zou, 2020). In the domain of language learning, its effectiveness in better learning performance was also evident in several studies, including listening comprehension (Hwang et al., 2017), business writing (Yu and Wang, 2016), vocabulary acquisition (Yu, 2014), and verb form learning (Yunus and Hua, 2021). Although translation competences differ from these sub-skills of language learning, the use of Quizizz appears to yield similar benefits and efficacy in translation training, as evidenced by the enhanced performance in translation tasks.

Although some previous studies did not find positive effects of using Quizizz in improving the learning outcome of language learners (e.g., Sénécal et al., 2022), the benefits of using this gamification application in the classroom are strongly supported by the current study and a wide range of related studies. The present study provides the confirmed support for the effectiveness and feasibility of this gamification tool to facilitate translation training, particularly for EFL learners with an intermediate level of English proficiency. This positive effect of response systems such as Quizizz may be explained by cognitive processes involved in translation training or learning. As noted by Yu (2014), Clicker-based instruction was quite useful to promote the vocabulary knowledge of EFL learners, mainly due to the dual functions of stimulating both deep and shallow semantic processing, effectively integrating long- and short-term memory to reinforce the memory of the words. This mechanism may similarly apply to translation, where linguistic competence, particularly the competence to understand the meaning in the source language and reformulate the meaning in another language, is a core component of proficient translation skills (Hurtado Albir, 2017).

Another possible factor contributing to the effectiveness of using Quizizz in improving the translation proficiency of the EFL learners may lie in the featured affordance of Quizizz to provide immediate feedback as to the learning performances of all the learners in the classroom. Once the students complete tasks assigned in class, both teachers and students can view performance results in real time. This timely feedback allows teachers to diagnose learning gaps, which makes a solid basis for further instruction adjustment. As a possible result, the teaching and learning in class could be more precisely targeted toward improving the proficiency of the learners. The better outcomes of translation proficiency observed in the Quizizz group may be attributed to this advantaged function inherent in gamified learning.

### 5.2 Effects of Quizizz on the engagement

With respect to research question 2, it was found that there was a lack of significant difference in post-test engagement scores between the Quizizz group and the control group. This suggests that the use of Quizizz did not produce the expected outcome in improving students' engagement in translation classroom. However, the Quizizz group, which initially had lower levels of engagement than the control group, demonstrated higher engagement levels in the post-test, even though the between-group difference did not reach the significant level.

This finding appears somewhat unexpected, as previous studies on gamification in learning have shown its positive effects in enhancing engagement, participation and motivation among learners across various contexts of academic subjects (e.g., Hung, 2018; Hwang et al., 2017; Seixas et al., 2016; Yu and Wang, 2016). However, a closer examination of these studies reveals that they focused on different gamification applications rather than Quizizz. For instance, the platforms employed in the study by Seixas et al. (2016) were two badging platforms that reward users with points. In the present study, Quizizz was used with mechanics and dynamics which may be different from the specific gamification systems used in their study. Functions of Quizizz used in the present study include immediate feedback, time limits on responses, point scores, and leaderboard ranking. These features may stimulate short-term affective reaction and motivate students' participation in classroom activities. However, such competitive features and instant feedback can increase short-term active involvement, translation learning or tasks still require maintained cognitive effort and strategic decision-making, which might impact their perceived engagement, particularly affective engagement over a longer period. Therefore, the effects of different gamification systems on learner engagement may be different, which may depend on how their mechanics are aligned with the cognitive demand of the skill areas or learning tasks. In this study, Quizizz may primarily support structured practice and feedback rather than producing noticeable changes in the perceived engagement level. Another possible explanation is that the translation learning, as the area of learning involved in this study, might require distinct engagement mechanisms compared to other subjects or even other language skills such as listening, writing and speaking. One critical factor in translation training is self-regulation strategies. As suggested by Pietrzak (2018), training on self-regulation strategies can enhance engagement in both the translation process and translation learning. This implies that engagement in translation learning may rely more on self-regulation strategies, which were not explicitly incorporated into the instructional design of Quizizz as adopted in this study.

This aligns with the suggestion from engagement studies (Hiver et al., 2021; Huang et al., 2020) that the unique aspects and features of different disciplines, instructional contexts, tasks and diverse groups of learners need to be accounted for in gamification across different educational settings. Futures studies could focus on exploring the engagement processes and strategies specific to translation training to gain deeper insights.

A third possible explanation for such an unexpected finding is that the initial levels of engagement among all the participants in this study were already high. The participants were learning English as their major of study and quite possibly, they may possess relatively higher levels of motivation and engagement in learning translation skills, which are essential skills for their future careers. As suggested by previous studies (e.g., Chen et al., 2023; Nelson et al., 2012), students with a relatively higher level of initial engagement may have a ceiling effect in the intervention so that the intervention of using Quizizz did not have a statistically significant effect on them, and instead it might work better on students of lower levels of engagement.

Finally, the lack of a significantly difference in engagement between the two groups may be related to how gamification was integrated into the teaching and learning process. As suggested by Alsawaier (2018) as to the effects of gamification on motivation and engagement, several guidelines need to be implemented to fully realize the positive effects, including appropriate time allocation on gamification, explicit learning objectives, reliable software, well-prepared questions, and opportunities for choice-making and collaboration. For Quizizz used in the present study, those features were not fully manifested in its design, which may have limited its impact on engagement. To interpret the different outcomes between students' translation proficiency and engagement as yielded in the present study, it may be possible that the affordances of gamification devices or platforms such as Quizizz, including immediate feedback, time-constrained decision making, and competitive ranking, may stimulate immediate strategic processing and problem-solving skills. These are central components of translation competence (Hurtado Albir, 2015). These mechanisms may primarily activate cognitive engagement and procedural knowledge development rather than affective engagement. Therefore, the overall improvement of engagement might not be as evident as that of translation proficiency.

### 5.3 Implications for research and pedagogical practice

These findings of this study have several implications for the research and teaching practice of technology-enhanced language learning classrooms. First, the use of gamification systems and applications such as Quizizz can play a facilitative role in improving learning outcomes in translation class or translation training. The more extensive use of gamification platforms or tools such as Quizizz should be encouraged in the teaching of translation courses focused on translation competence training. Future studies could examine and compare the effects of different types of gamification tools to provide more insightful and comprehensive understanding given the insufficient research efforts on gamifying the translation classroom.

Secondly, while Quizizz effectively enhanced translation proficiency, it did not seem to significantly improve the perceived engagement among the students. Therefore, strategies to foster engagement need to be explored in-depth to expand the understanding of the specific process of promoting learner engagement in translation training. Other factors such as learners' existing level of engagement and translation competence, as well as psycho-cognitive processes such as self-regulatory strategies, warrant closer investigation due to their potential critical roles in enhancing the engagement.

Thirdly, gamifying the translation classroom is recommended to be implemented with a wider scope so as to produce beneficial outcomes in elevating students' level of engagement. Only partially integrating gamification into traditional teaching may not yield the expected engagement improvements. Gamification should be holistically incorporated into all aspects and procedures of teaching, including in-class instruction and learning, after-class learning, assignment, and assessment as well.

For pedagogical practice in translation classroom, the findings suggest that gamified educational systems such as Quizizz may achieve better outcomes when they are appropriately integrated into translation instruction, instead of being used merely for entertaining or competitive features. Instructors may use gamification tools to create short retrieval-based activities for the purpose of enhancing learners' mastery of main translation strategies, facilitating their notice on language differences, and improving their problem-solving or decision-making skills. They can utilize the immediate feedback provided by the system to diagnose common issues or errors and encourage students to reflect on their decisions before moving on to more complete translation tasks. In addition, instructors can use Quizizz in a formative way, for example, collecting anonymous responses, comparing different versions of translation, and directing the brief class discussions. The use of gamified tools should place more emphasis on cognitive and strategy development, instead of being simply driven by novelty or affective arousal. Given the substantial effect in improving translation proficiency whereas no obvious change in engagement, it is proposed that diverse activities should be designed and used to promote engagement, for example, formative gamified micro-tasks, immediate feedback cycles, and structured scaffolded strategy reinforcement such as group or pair discussions, in-depth reflections or discussions on different translations, and peer feedback on students' translation drafts.

## 6 Conclusions

Gamification applications such as Quizizz can be utilized in the proper way to benefit the language classroom, in the case of the present study, the translation classroom. These findings contribute to the broader research on the effectiveness of gamification in pedagogical practice, reinforcing the affordances of gamified teaching approaches in education. The better outcomes of translation learning as shown by this study make a solid case for using gamified teaching mode to benefit translation instruction or training.

As for the engagement of the learners, although the engagement might not benefit as much as the learning outcome, future studies should explore engagement processes in other language skills and investigate the unique features and mechanisms of engagement in translation learning or training. In addition, this study has its limitations, including a homogeneous participant sample, non-random group assignment, and a relatively short period of intervention. The quasi-experiment design with two intact classes selected might incur cluster effects which may have influenced the independence of observations. Given that only two classes were included, multi-level modeling was not statistically feasible in this case. Although ANCOVA was conducted to control for baseline differences, the differences in translation baseline scores between the two groups remain a limitation of the study. To better account for class-level variance and strengthen causal inference, future studies with a larger number of clusters and randomized assignment of individual participants are recommended to address these limitations and examine how utilization of gamified learning in the translation classroom may impact other processes or learner variables such as, but not exclusive to, self-efficacy, learning goals, self-regulation or self-autonomy, anxiety, motivation and interest. Pedagogical and research endeavors in gamified translation education can contribute to fostering more autonomous, effective and engaging learning experiences for translation learners.

## Data Availability

The raw data supporting the conclusions of this article will be made available by the authors, without undue reservation.
