# Supplementary material for: Effects of gamification on the engagement and translation proficiency of the Chinese EFL learners: a case of Quizizz
Source: Front Psychol. 2026 Apr 13;17:1813663. doi: 10.3389/fpsyg.2026.1813663 (PMC13111394; doi:10.3389/fpsyg.2026.1813663)
Supplement: Supplementary file 1 [file Supplementary_file_1.pdf]

Appendix

Week 1	Pre-test of translation and course information
Week 2-3	General introduction of translation: History, criteria, process and basic qualifications
Week 4	Comparison of the Chinese and English languages
Week 5	Comparison of the Chinese and English cultures
Week 6	Translation of words
Week 7	Translation of comparative, emphatic and inverted sentences
Week 8-10	Translation techniques
Week 11	Translation of attributive sentences
Week 12	Translation of adverbial sentences
Week 13	Translation of long sentences
Week 14	Post-test of translation

Adapted from Khanmohammad & Osanloo (2009)

Score range	Description
	Accuracy (30%)
25-30	No identifiable problems of comprehension;<br>original message has been conveyed completely to TL readers;<br>no omissions or additions to information
21-24	Virtually no problems of comprehension except with the most

	highly specialized vocabulary with no influence on TL readers
16-20	Information is conveyed to TL readers with some difficulty due<br><br>to translator misunderstanding of some parts of original message; apparent omissions and additions
11-15	Poor expression of ideas;<br><br>numerous serious problems in understanding ST interfere with communication of original
1-10	Severe problems interfere greatly with communication of original message;<br><br>TL reader can't understand what original writer was trying to say
Finding equivalent (25%)	
20-25	All lexical and syntactic elements have been understood;<br><br>precise vocabulary usage; words have been chosen so skillfully that the work reads like a good publishable version
15-19	Full comprehension and good usage of a wide range of<br><br>vocabulary and structures;<br><br>specialized vocabulary presents some problems with unsuitable equivalents
10-14	General comprehension of a fair range of vocabulary although some gaps observed;<br><br>some vocabulary misused;<br><br>some evidence of plausible attempts to work around difficulties of<br><br>finding equivalents, perception, wordplay and other linguistic features
5-9	Comprehension of vocabulary and structures show quite noticeable gaps which obscure sense;<br><br>problems in finding correct vocabularies;

	unable to cope with specialized vocabulary
1-4	Inappropriate use of vocabularies;<br><br>comprehension of original seriously impeded even with fairly everyday vocabulary and structures;<br><br>translation as a whole makes little sense
Register, TL culture (20%)	
17-20	Good sensitivity to nuances of meaning, register are precisely and sensitively captured；<br><br>there is a sophisticated awareness of the cultural context;<br><br>translation shows a sophisticated command of TL lexis, syntax, and register
13-16	There is a fair degree of sensitivity to nuances of meaning, register, and cultural context
9-12	There is a lack of sustained attention to nuances of meaning, register, and cultural context; no awareness of register; TL lexis, syntax, and register are not always appropriate
4-8	There is scant attention to nuances of meaning, register, and cultural context;<br><br>there are serious to severe shortcomings in the use of appropriate lexis, syntax, and register
1-3	There is no appreciable understanding of nuances of meaning, register, and cultural context;<br><br>no concept of register or sentence variety
Grammar and ST style (15%)	
13-15	Gives the feeling that the translation needs no improvement from grammatical and stylistic points though one or two natural failings might be observed;

	native-like fluency in grammar
10-12	Shows flair for stylistic manipulation of TL items as if text were written in TL originally except where the language is placed under severe pressure of comprehension;<br>maintains advanced proficiency in grammar,<br>some grammatical problems but with no influence on message
7-9	Tends to have awkward grammatical usage in TL and literality of rendering though but not impeding sense in a significant manner; some attempts to reflect stylistic features of the original; some grammatical problems are apparent and have negative effects on communication
4-6	Clumsy TL; often nonsensical grammatical usages in TL;<br>unnatural sounding;<br>little attempt to reflect stylistic features of the original;<br>there is evidence of clear difficulties in following
Shifts, omissions, additions and inventing equivalents (10%)	
9-10	Correct use of relative clauses, verb forms;<br>use of parallel structure;<br>creative inventions and skillful solutions to equivalents;<br>no fragment or run-on sentence
7-8	Almost all shifts appear with partial trespass, attempts variety;<br>some inventions for not available equivalents in TL;<br>no fragment or run-on sentence
5-6	Some shifts but not consistency;<br>awkward and odd structure;<br>only few run-on sentences or fragments present

3-4	Lacks variety of structure due to not preserving necessary shifts except for few cases;<br><br>little or no evidence of invention in equivalents
1-2	Unintelligible sentence structure due to completely ignoring necessary shifts;<br><br>no skillful handling of equivalents;<br><br>no trace of invention
